# Polysubstance use in adolescents: conceptual issues and the need for a new public health framework and approach

**DOI:** 10.1017/S0033291726104504

**Published:** 2026-05-14

**Authors:** Ronan Fleury, Lorna Staines, Bobby Smyth, Mary Cannon

**Affiliations:** 1 Department of Psychiatry, RCSI University of Medicine and Health Sciences, Dublin, Ireland; 2 Department of Psychology, National College of Ireland Maynooth, Maynooth, Kildare, Ireland; 3 Department of Public Health Northwest, Health Service Executive, Sligo, Ireland; 4 Department of Public Health & Primary Care, Trinity College Dublin, Dublin, Ireland; 5 Future Neuro Research Ireland Centre, RCSI, Dublin, Ireland

**Keywords:** adolescences, polysubstance use, public health, substance use

## Abstract

Polysubstance use is increasingly the norm among adolescents who use substances, yet research and clinical frameworks remain concentrated in single-substance models. This narrative conceptual editorial highlights key emerging literature on the conceptual inconsistencies and methodological challenges in polysubstance use research, particularly in the context of shifting cannabis legislation and adolescent vulnerability. A lack of definitional consensus, regarding the number of substances, timing of engagement, and intent of substances used, undermines the comparability of findings and the effectiveness of interventions. Adolescent polysubstance use is shaped by developmental, social, and structural factors, with cannabis and nicotine frequently overlooked despite their prevalence. This editorial addresses three linked gaps: definitional inconsistencies, insufficient attention to timing and intent of co-use, and the continued dominance of single-substance research frameworks. Therefore, we argue for clearer definitions, inclusive research designs, and interdisciplinary approaches.

In recent years, research and clinical exploration of substance use have been forced to confront a complex and evolving reality: that single-substance frameworks no longer reflect the lived experiences of many people who engage in substance use, particularly adolescents. Recent evidence suggests that adolescent polysubstance use is substantial, though estimates vary by sample, age, and definition. In a 2021 school-based sample of 15- to 16-year-olds, Fleury et al. (2026) reported that 7.5% had used nicotine, alcohol, and cannabis in the past 30 days. By age 20 years, this figure has been reported to rise to 43% for the same substance combination (Brennan et al., 2025). Earlier European data also indicate notable levels of adolescent polysubstance use, with nearly 30% of 15- to 16-year-olds across 22 countries reporting use of multiple substances (EMCDDA, 2009). As public health initiatives try to catch up with the multifaceted nature of polysubstance use, a range of definitional, methodological, and cultural challenges have appeared. These challenges are further intensified by the shifting legal status of cannabis across jurisdictions, often outpacing both the research infrastructure and clinical preparedness needed to respond.

The issue of polysubstance use, *broadly* defined as the use of more than one substance, whether simultaneously or concurrently (i.e. within the same occasion or across a defined period of time), has emerged to the forefront of multiple disciplines, including addiction science, public health, psychology, and psychiatry (Green, 2023). Yet, as Bunting et al. (2024) argue, despite the term’s increasing presence in public health-related documents and research grant proposals, its precise meaning remains *frustratingly* ambiguous. Different disciplines (clinicians and researchers) define polysubstance use variously by the number of substances, the timing of use, or the presence of multiple substance use disorders. In the absence of clear standards, comparisons across studies become diluted, undermining efforts to synthesize findings and guide clinical care. For example, adolescents who use substances simultaneously may require different prevention and clinical responses than those whose use overlaps across a broader time period, as the immediate risks, motivations, and associated harms may differ.

Nowhere is this lack of clarity more damaging than in research with adolescents, a population in which substance use is shaped by neurodevelopment, social pressures, and experimentation. Green (2023), in a succinct but urgent letter to the editor, highlights the diversity of definitions applied in recent adolescent studies, ranging from multi-year windows of use to same-day co-ingestion, calling attention to how such variation obstructs the development of appropriate interventions. Without consensus on what we are measuring, how can we expect to make meaningful recommendations for prevention or treatment?

However, this is not just an academic concern. As Crummy, O’Neal, Baskin, and Ferguson (2020) note, polysubstance use is now understood to be predictive of significantly worse outcomes. These include higher rates of treatment dropout, relapse, and mortality, particularly when opioids are part of the mix (Kelleher, Riordan, and Lyons, 2024). Research by Philbin and Mauro (2019) shows empirical evidence that polysubstance use patterns are often tracked along life-course trajectories, with patterns of use that evolve, stabilize, or escalate over years, sometimes quietly, and are shaped by structural inequities.

Much of the problem reflects how substance use research frameworks were originally constructed. That said, some longitudinal and life-course evidence to date has begun to examine more complex polysubstance use patterns, particularly in adult populations (Brennan et al., 2025; Steinhoff et al., 2022). As Hakkarainen, O’Gorman, Lamy, and Kataja (2019) argue, the dominant models of substance use, especially those developed within neuroscience and epidemiology, have historically aimed for simplicity and control, for example, a single substance-use model. Participants who use multiple substances are often excluded from clinical trials in favor of ‘*clean*’ data, leaving us with findings that do not reflect the reality of the people we are trying to help. As a result, we risk designing interventions that are both irrelevant and ineffective.

Indeed, one of the most consistent findings across the literature is what we are failing to measure. Tobacco use, for instance, is frequently omitted or presumed. As Bunting et al. (2024) point out, nicotine is not only a common companion to other substances but may itself act as a potentiator, modifying the effects of other drugs and complicating withdrawal and treatment dynamics. Yet in many studies of illicit drug use, tobacco is excluded from analysis, perhaps in part due to its legal status.

We would also argue that a key area of polysubstance use is not currently being considered; intent in polysubstance use remains critically underexplored. Whether substances are used simultaneously by design (e.g. to enhance or balance effects) or incidentally (e.g. through social availability, habit, misingestion, or overlap) has profound implications for both risk profiles, intervention strategies, and public-health policy. For example, a teenager who combines cannabis and alcohol to intensify intoxication, for instance, may require different clinical engagement than one who drinks socially and happens to smoke. Yet few studies differentiate deliberate co-use from incidental or deliberate overlap, and intent is rarely captured in standard survey instruments or diagnostic criteria for substance use/polysubstance use disorders. Without accounting for why substances are used together, we risk flattening the nuanced realities of use and overlooking opportunities for targeted prevention and public-health strategies.

The legalization and normalization of cannabis adds a further layer of complexity. While cannabis has long been present in polysubstance combinations, frequently with alcohol, nicotine, or stimulants, it is now increasingly perceived as harmless or even therapeutic by adolescents (O’Dowd et al., 2025). The data strongly contradict this perception. Cannabis use, especially when used with alcohol, is associated with increased risk behaviors, including unsafe driving, heavier overall substance use, and a higher likelihood of substance use disorders and psychosis (Smyth, Fleury, and Cannon, 2024). Yet cannabis is rarely the primary focus in polysubstance research. It is often treated as peripheral or excluded from analysis altogether (Bunting et al., 2024), despite real-world data showing its frequent co-use with alcohol and nicotine among adolescents (Fleury et al., 2026).

**What, then, are the implications for researchers and clinicians, particularly in countries like Ireland and the United Kingdom, where cannabis policy is in flux and adolescent mental health services are under pressure?**

First, we need definitional clarity. Drawing on existing recommendations, we propose that polysubstance use be defined by three core dimensions: (1) the number and type of substances used (e.g. specific substances or classes such as alcohol, nicotine, cannabis or stimulants and sedatives); (2) the timing of use (with categories such as simultaneous, same-day, or sequential); and (3) the intent of use, including whether the combination was deliberate or incidental overlapping. These dimensions should be operationalized consistently across studies and clearly reported in both clinical and public health research.

Second, we must stop excluding the very populations most at risk. Evidence from adult cohorts suggests that polysubstance use can follow distinct trajectories over time (Steinhoff et al., 2022). However, adolescent substance use patterns may differ due to developmental processes, peer contexts, and psychosocial strategies (O’Dowd et al., 2025). Adolescents who use multiple substances should not be omitted from studies simply because they complicate analyses. Instead, we need to develop tools, both quantitative and qualitative, that can capture the heterogeneity of their use patterns. For example, epidemiological surveys should routinely record whether substances were used simultaneously or concurrently, while including nicotine and cannabis. Furthermore, this includes investing in preclinical models that reflect polysubstance conditions and designing longitudinal studies that follow trajectories of use over time.

Third, cannabis must be foregrounded, not backgrounded. As its legal and social status changes, so too will its role in polysubstance patterns. Researchers should avoid the trap of treating cannabis as a ‘neutral’ comparator or as somehow less important than opioids or stimulants. Its use, especially when initiated in adolescence, is independently associated with harms, and when co-used with other substances, those harms only multiply (Fleury et al., 2026; Hurd, 2025). Clinicians should consider overlap in substance use and question simultaneously or concurrently engagement experiences during intake.

Finally, we need to rethink our metrics of harm. While overdose remains a critical endpoint, it is not the only one. Academic decline, social disconnection, criminalization, and mental health deterioration are all part of the polysubstance use landscape (Brennan et al., 2024, 2025; Power et al., 2021). These outcomes are particularly salient in adolescence, where patterns established early can shape entire adult lives. Without these changes, research risks continuing to misclassify real-world patterns of use, clinical assessments may miss important features of adolescent substance use, and prevention efforts may poorly match the behaviors they are intended to address.

It is time to embrace the full picture: complex, contextual, and often contradictory. Polysubstance use is not the exception; it is the norm among people who engage in substance use. Our research, policy, and clinical care must evolve accordingly.
